# Icariin accelerates cartilage defect repair by promoting chondrogenic differentiation of BMSCs under conditions of oxygen‐glucose deprivation

**DOI:** 10.1111/jcmm.17073

**Published:** 2021-12-03

**Authors:** Wang Tang, Hongyi Zhang, Donghua Liu, Feng Jiao

**Affiliations:** ^1^ Spinal Surgery Guangzhou Hospital of Integrated Traditional and Western Medicine Guangzhou China; ^2^ Joint Surgery Guangzhou Hospital of Integrated Traditional and Western Medicine Guangzhou China

**Keywords:** bone marrow mesenchymal stem cells, chondrogenic differentiation, Icariin, proliferation

## Abstract

This study explored the role played by combined ICA and bone mesenchymal stem cells (BMSCs) in repairing rabbit knee cartilage defects. Firstly, rabbit BMSCs were isolated and used to construct an in vitro cellular model of oxygen‐glucose deprivation/reoxygenation (OGD/R). Subsequently, ICA processing, Alcian blue staining, immunofluorescence and Western blot studies were performed to evaluate the ability of BMSCs to display signs of chondrogenic differentiation. Furthermore, a rabbit knee cartilage injury model was established in vivo. International Cartilage Repair Society (ICRS) macroscopic evaluations, H&E, Alcian blue and EdU staining, as well as immunohistochemistry, were analysed cartilage repair and pathological condition of the knee cartilage tissue. Our in vitro results showed that ICA promoted the chondrogenic differentiation of BMSCs, as well as aggrecan (AGR), bone morphogenetic protein 2 (BMP2) and COL2A1 protein expression in BMSCs. In vivo experiments showed that rabbits in the BMSCs or ICA treatment group had higher ICRS scores and displayed a better restoration of cartilage‐like tissue and chondrocyte expression on the surface of their cartilage defects. In conclusion, ICA or BMSCs alone could repair rabbit knee cartilage damage, and combined treatment with ICA and BMSCs showed a better ability to repair rabbit knee cartilage damage.

## 1 INTRODUCTION

Osteoarthritis is a degenerative joint disease in which the main pathological change is the destruction of articular cartilage^1^, ^2^ that serves to bear weight and relieve contact stress.^3^ Articular cartilage is particularly susceptible to damage because it lacks neurons and blood vessels, making it difficult to repair itself once damage occurs.^4^, ^5^ Microfracture and chondrocyte transplantation are often used clinically to repair damaged cartilage tissue^6^; however, the long‐term satisfaction with the curative effect of this procedure is low, and large amounts of fibrocartilage components appear in the newly formed cartilage tissue after the operation, which affects the flexibility of the joints after microfracture.^6^ Matrix‐induced autologous chondrocyte implantation is a reliable method for treating a cartilaginous defect; however, the financial cost of the procedure is high.^7^ Hence, there remains a need to develop more effective methods for treating cartilage damage.

Mesenchymal stem cells exhibit the ability to differentiate into specific cell types and self‐renew and therefore play a key role in tissue repair.^8^ Research has shown that mesenchymal stem cells extracted and expanded from bone marrow can promote the regeneration of bone and cartilage,^9^, ^10^ and immune regulation,^11^ which prevents further cartilage damage. Bone marrow mesenchymal stem cells (BMSCs) have a paracrine function that regulates surrounding cells and are beneficial to the synthesis of cartilage matrix.^12^ A recent study showed that BMSC‐assisted injectable Collagen I hydrogel can regenerate a cartilage defect and remould cartilage homeostasis.^13^ However, when BMSCs are transplanted into a region of ischaemic and hypoxia tissue, the survival rate of the BMSCs is reduced, and their repair ability is also impaired.^14^ Therefore, enhancing the tolerance of BMSCs to ischaemia and hypoxia, as well as improving graft survival are essential for achieving cartilage repair.

Icariin (ICA) is a flavonoid glycoside compound and effective pharmacological ingredient found in the traditional Chinese medicine Epimedium.^15^ ICA has anti‐osteoporosis, anti‐inflammation, anti‐tumour and anti‐oxidative stress effects and also helps to protect cardiovascular function.^16^ ICA has been widely used for treating osteoporosis^17^ and also in experimental research on osteoblasts^18^ and osteoclasts.^19^ In addition, ICA can suppress the lipopolysaccharide‐induced inflammation of chondrocytes.^20^ All these studies suggest the role of ICA on cartilage tissue engineering. The introduction in the previous paragraph has demonstrated the promoting effects of BMSCs on the regeneration of bone and cartilage. However, the therapeutical effects of BMSCs are discounted due to restricted survival time of BMSCs. Interestingly, previous study has shown that ICA can induce the synthesis of alkaline phosphatase and formation of calcified nodules to promote the differentiation of rat BMSCs into osteoblasts.^18^ Moreover, ICA has been reported to exert anti‐apoptosis effect on various cells.^21^, ^22^, ^23^ Recent study has also proved that ICA has the potential to induce BMSC proliferation and differentiation into chondrocytes and protect rabbit BMSCs against oxygen‐glucose deprivation (OGD)‐induced apoptosis.^24^ Thus, we speculated whether the combination of ICA and BMSCs shows the superior effects on cartilage defects in comparison with ICA or BMSCs alone. Most of studies have explored the in vitro effects of ICA on chondrocytes; however, the in vivo experiments on the role of combined ICA and BMSCs in repairing rabbit knee cartilage defects are lacking. Therefore, we established both an oxygen‐glucose deprivation/reoxygenation (OGD/R) BMSC model and a rabbit knee cartilage injury model and used them to explore the effects of ICA and BMSCs in treating cartilage injuries at the cellular level in vitro and animal level in vivo.

## 2 MATERIALS AND METHODS

### 2.1 Cell isolation and culture

New Zealand rabbits (1‐month old) were purchased from the animal holding unit of the Fourth Military Medical University. BMSCs were isolated from rabbit bone marrow as previously described.^25^, ^26^ In brief, samples of bone marrow were cultured in DMEM (Gibco, Waltham, MA, USA) containing 100 U/ml penicillin/streptomycin, 272 μg/ml L‐glutamine and 10% foetal bovine serum (FBS, Gibco). The medium was changed every three days.

### 2.2 Stem cell characterization assay

BMSCs isolated from bone marrow were cultured to passage 3 and subsequently cultured for osteogenic, chondrogenic and adipogenic differentiation to explore the differentiation characteristics of stem cells.^24^, ^27^ For analysis of osteogenic differentiation, BMSCs were induced and then cultured in osteogenic induction medium at a density of 3 × 10^3^ cells/mL for 14 days; after which, they were fixed with 4% paraformaldehyde for 30 min and stained with Alizarin red S (Sigma‐Aldrich, St. Louis, MO, USA) for 15 min. Similarly, for analysis of adipogenic differentiation, BMSCs were cultured in adipogenic induction medium for 14 days and then stained with 0.3% Oil Red O (Cyagen Biosciences, Santa Clara, CA, USA) for 30 min. For the analysis of chondrogenic differentiation, BMSCs were induced and cultured in chondrogenic induction medium at a density of 3 × 10^3^ cells/ml for 14 days. After removal of the chondrogenic induction medium, the cells were fixed with 4% paraformaldehyde for 30 min and then washed twice with phosphate buffered saline (PBS). The cells were then incubated with 1% Alcian blue 8GX (Sigma‐Aldrich) for 1 h at room temperature. Staining results were observed under a confocal microscope (Nikon EclipseTS100, Tokyo, Japan).

### 2.3 Cellular oxygen and glucose deprivation treatment

BMSCs were cultured in glucose‐ and serum‐free DMEM within an oxygen‐free incubator at 37°C for 4 h and then reoxygenated in DMEM medium for use in further experiments. To evaluate the effect of ICA on oxygen‐glucose deprivation/reoxygenation (OGD/R)‐induced BMSCs, 0.1, 1 or 10 µM ICA (PureOne Biotechnology, Shanghai, China) was added to the medium at 24 h before OGD/R conditions were applied. The cells were divided into five groups that included a Control, OGD/R, OGD/R + 0.1 µM ICA, OGD/R + 1 µM ICA and OGD/R + 10 µM ICA group. Simultaneously, the cells were also cultured in chondrogenic induction medium for subsequent analysis of their chondrogenic ability by Alcian blue staining.

### 2.4 Immunofluorescence

Immunostaining was performed to analyse the expression of bone morphogenetic protein 2 (BMP2), aggrecan (AGR) and collagen type II alpha 1 (COL2A1) in BMSCs after 3 days, 7 days and 14 days of incubation in chondrogenic differentiation medium. The cells were fixed with 4% paraformaldehyde at room temperature for 30 min, permeabilized with 0.1% Triton X‐100 and then blocked with 1% bovine serum albumin in PBS. Next, the cells were incubated with rabbit polyclonal anti‐BMP2, AGR and COL2A1 antibodies (Abcam, Cambridge, MA, USA) overnight at 4°C and subsequently incubated with a CM‐Dil labelled goat anti‐rabbit secondary antibody. In addition, the actin cytoskeleton was stained for 45 min with phalloidin‐Atto488 (Sigma‐Aldrich), and BMP2 and AGR markers were also stained in the cells. After washing with PBS, the nuclei were stained with 4′,6‐diamidino‐2‐phenylindole (DAPI) (Sigma‐Aldrich), and staining results were observed under a confocal microscope (Nikon EclipseTS100, Tokyo, Japan).

### 2.5 Alcian blue staining

Alcian blue is dissolved in 0.5 mol/L sodium acetate solution at the rate of 1.4 mg/ml. 100 µl standard solution containing 10, 20, 40, 60 and 80 μg chondroitin sulphate was added to the test tube, 100 μl deionized water was added to the blank control, 1.5 mlof Alcian blue solution was added to each test tube, and it was detected at 490 nm by enzyme‐linked immunosorbent assay. The absorbance value of cell supernatant in each group was measured by the same method, and the content of GAGs in cell solution was obtained according to the standard curve.

### 2.6 DMMB assay of GAG

BMSCs were cultured in glucose‐ and serum‐free DMEM at 37°C for 24 h. After incubating for 24 h, the conditioned medium was collected for GAG synthesis analysis. Conditioned medium was collected from each experimental group, and the presence of GAG released from primary chondrocytes was quantified using dye DMMB. Culture medium was pretreated with 0.5 units/ml of hyaluronidase at 37 °C for 3 h in order to get rid of exogenous HA in order to remove interference. Digests were mixed with DMMB in 96‐well plates and read at 520 nm with Spectra Max 384 Microplate Reader.

### 2.7 Western blotting

The levels BMP2, AGR and COL2A1 (1:1000, Abcam, Cambridge, USA) protein expression in BMSCs after 3 days, 7 days and 14 days of incubation in chondrogenic differentiation medium were further analysed by Western blotting. BMSCs were lysed in RIPA cell lysis buffer containing a protease inhibitor, and the amount of total protein in each sample was detected using a BCA protein assay kit (Solarbio, Beijing, China). Next, a 50 µg aliquot of total protein from each sample was separated by 12% SDS‐PAGE, and the protein bands were transferred onto polyvinyliene difluoride (PVDF) membranes (Millipore, Burlington, MA, USA). The membranes were blocked with 5% skimmed milk for 2 h at room temperature and then incubated overnight with primary antibodies 4°C, followed by incubation with a horseradish peroxidase (HRP)‐conjugated secondary antibody at room temperature for 2 h. Next, the membranes were washed 3 times with TBST and visualized with an enhanced chemiluminescence system (Merck Millipore, Darmstadt, Germany). The signal intensity in the films was analysed with an Alphalmager HP system (Cell Biosciences, Inc., Santa Clara, CA, USA).

### 2.8 Construction of the articular cartilage injury model

A total of 84 six‐month‐old New Zealand white rabbits (2.5 kg each) were used in this study. The growth environment temperature of all animals is maintained at 22°C, and the relative humidity is 55%. The sterilized special feed was provided, and the food and water resources were freely obtained. The rabbits were anaesthetized by intravenous injection of 3% pentobarbital (40 mg/kg) supplemented with a subcutaneous injection. Next, each anaesthetized rabbit was fixed on an operating table in the supine position, the hair around the right knee joint was shaved with a razor, and the right knee joint was wiped with iodophor. The right knee joint was covered with a hole towel to expose the knee surgery area. The knee joint was bent along the inner side of the patellar ligament, and a 3.0–4.0 cm incision was made on the inner side of the knee joint patellar ligament. Next, the patella was pushed to the outer side to expose the femoral trochlear. A hand drill was used to create a cartilage defect on the trochlear surface of the femur. The trochlear surface of the femur was positioned with a 3 mm drill bit, and a hole with a diameter of 3 mm and a depth of 4 mm was drilled. PBS was used to wash away tissue debris and blood clots. Finally, the patella was reset, and the wound was sutured and disinfected with iodophor. Penicillin (800,000 U) was continuously injected into the breech muscle each day for 3 consecutive days after the operation. This protocol for this study was approved by the Institute Animal Care and Use Committee of Guangzhou Hospital of Integrated Traditional and Western Medicine (Guangzhou, Guangdong, China). This study followed the ARRIVE guidelines.

### 2.9 ICA and BMSC processing

The above animal models were randomly divided into 4 groups with 7 rats in each group. These models included an Operation (Operation), Operation + BMSCs (BMSCs), Operation + ICA (ICA) and Operation + ICA + BMSCs (ICA + BMSCs) group. The specific treatment process was as follows. 8 × 10^5^ U of penicillin was injected intramuscularly into the breech for 3 days after modelling. In the Operation group, the joint cavity was injected with an equal volume of saline (1 ml). In the BMSCs group, the joint cavity of the rabbit was injected with 1 ml of rabbit BMSCs (1 × 10^7^ cells) in the first two treatments. In the third treatment, the joint cavity was injected with 1 mL of rabbit BMSCs (1 × 10^7^ cells) that had been incubated with 10 μM EdU for 48 h in advance. In the ICA group, 1 ml of 10 μM ICA was injected into the ear vein for a total of three administrations. In the ICA + BMSCs group, the joint cavity of the rabbit was injected with 1 ml of rabbit BMSCs (1 × 10^7^ cells) in the first two treatments, and in the third treatment, the joint cavity was injected with 1 ml of rabbit BMSCs (1 × 10^7^ cells) that had been incubated for 72 h with 10 μM ICA in advance and also incubated with 10 μM EdU for 48 h. Then, after the last treatment, rabbits were observed for 4 weeks, 8 weeks and 12 weeks respectively (Table 1).

**TABLE 1:** Treatment process for rabbit

Times	1 w	3 d (once a day)	1 w (once a week)	1 w (once a week)	1 w (once a week)
Group					
Operation (21)	Adaptive feeding	Modelling + Penicillin (8 × 10^5^ U)	1 ml saline^a^	1 ml saline^a^	1 ml saline^a^
BMSCs (21)	Adaptive feeding	Modelling + Penicillin (8 × 10^5^ U)	1 ml BMSCs^b^	1 ml BMSCs^b^	1 ml BMSCs^b^
ICA (21)	Adaptive feeding	Modelling + Penicillin (8 × 10^5^ U)	1 ml 10 μM ICA^c^	1 ml 10 μM ICA^c^	1 ml 10 μM ICA^c^
ICA + BMSCs (21)	Adaptive feeding	Modelling + Penicillin (8 × 10^5^ U)	1 ml BMSCs (incubated with 10 μM ICA)^d^	1 ml BMSCs (incubated with 10 μM ICA)^d^	1 ml BMSCs (incubated with 10 μM ICA)^d^

^a^1 ml saline was injected into the joint cavity

^b^1 ml rabbit BMSCs (a density of 1 × 10^7^) incubated with 10 μM EdU for 48 h were injected by joint cavity

^c^1 ml ICA (10 μM) was injected into the ear vein

^d^1 ml rabbit BMSCs (a density of 1 × 10^7^) incubated with 10 μM ICA for 72 h and 10 μM EdU for 48 h were injected by ear vein or joint cavity.

### 2.10 ICRS

At 4, 8 and 12 weeks, respectively, 5 rabbits in each group were sacrificed by anaesthesia. The whole knees of the rabbits were dissected, and the distal femur was removed. Cartilage damage was assessed based on the International Cartilage Repair Society (ICRS) gross morphology assessment scale for cartilage repair as shown in Table 2. And three scientists performed the scoring, and it was treatment blinded.

**TABLE 2:** International Cartilage Repair Society macroscopic evaluation of cartilage repair

Feature	Score
*Degree of defect repair*	
In level with surrounding cartilage	4
75% repair of defect depth	3
50% repair of defect depth	2
25% repair of defect depth	1
0% repair of defect depth	0
*Integration to border zone*	
Complete integration with surrounding cartilage	4
Demarcating border <1 mm	3
3/4 of graft integrated, 1/4 with a notable border >1 mm width	2
1/2 of graft integrated with surrounding cartilage, 1/2 with a notable border >1 mm	1
From no contact to 1/4 of graft integrated with surrounding cartilage	0
*Macroscopic appearance*	
Intact smooth surface	4
Fibrillated surface	3
Small, scattered fissures or cracks	2
Several, small or few but large fissures	1
Total degeneration of grafted area	0
*Overall repair assessment*	
Grade I: normal	12
Grade II: nearly normal	11–8
Grade III: abnormal	7–4
Grade IV: severely abnormal	3–1

### 2.11 Pathological analysis

The lower ends of the femurs were fixed with paraformaldehyde for 1 day and then decalcified in 10% EDTA (pH 7.3) for 21 days.^28^ Sections (5 μm thick) were cut in the transverse plane through the central part of the femoral trochlea. The sections were then stained with haematoxylin for 5 min and eosin for 2 min, washed with water, dehydrated with gradient alcohol, treated with xylene twice for 5 min each and sealed with neutral resin. The prepared sections were observed under an optical microscope. Other sections were treated with a descending ethanol series, dewaxed and stained with 1% toluidine blue at room temperature for 3 min. Those staining results were also observed under an optical microscope.

### 2.12 Immunohistochemistry

After tissue decalcification and paraffin embedding, immunohistochemistry was performed.^29^ The sections were incubated with rabbit anti‐COL2A1 antibody at 4°C overnight and subsequently incubated with the secondary goat anti‐rabbit antibody labelled with horseradish peroxidase for 30 min at 37°C. The colour reaction was developed with 3, 3′‐diaminodenzidine, and the tissue sections were counter‐stained with haematoxylin. The prepared sections were observed under an optical microscope.

### 2.13 Cartilage repair ability analysis

In order to further analyse the repair of cartilage in the cartilage damage area, EdU staining was performed to analyse cell proliferation occurring in the damaged tissue. Tissue sections were blocked with 1% bovine serum albumin in PBS for 1 h and then incubated with anti‐EdU rabbit antibody (Sigma‐Aldrich) overnight at 4°C. Next, the tissue sections were washed 3 times with PBS and then incubated with HyLyte Fluor 555 labelled anti‐rabbit antibody at room temperature for 1 h. Nucleic acids were stained with DAPI (Sigma‐Aldrich) for 5 min. Finally, the proliferative ability of the tissue cells was evaluated under a confocal microscope (Nikon EclipseTS100). The staining intensity score was determined as 0 = negative, 1 = weak, 2 = moderate and 3 = strong. The positive rate score was determined as 0 = negative, 1 = (1–25%), 2 = (26–50%), 3 = (51–75%) and 4 = (76–100%). EdU scores superior to 6 in cancer tissues were defined as “Completely repair”.

### 2.14 Statistical analysis

All experiments were repeated three times, and the data were analysed using GraphPad Prism 8.0.2 software. Differences between groups were analysed by one‐way or two‐way analysis of variance (ANOVA), followed by Tukey’s multiple comparisons test for multiple comparisons. A *p*‐value < .05 was considered to be statistically significant.

## 3 RESULTS

### 3.1 Isolated BMSCs displayed the differentiation potential of stem cells

**FIGURE 1:**
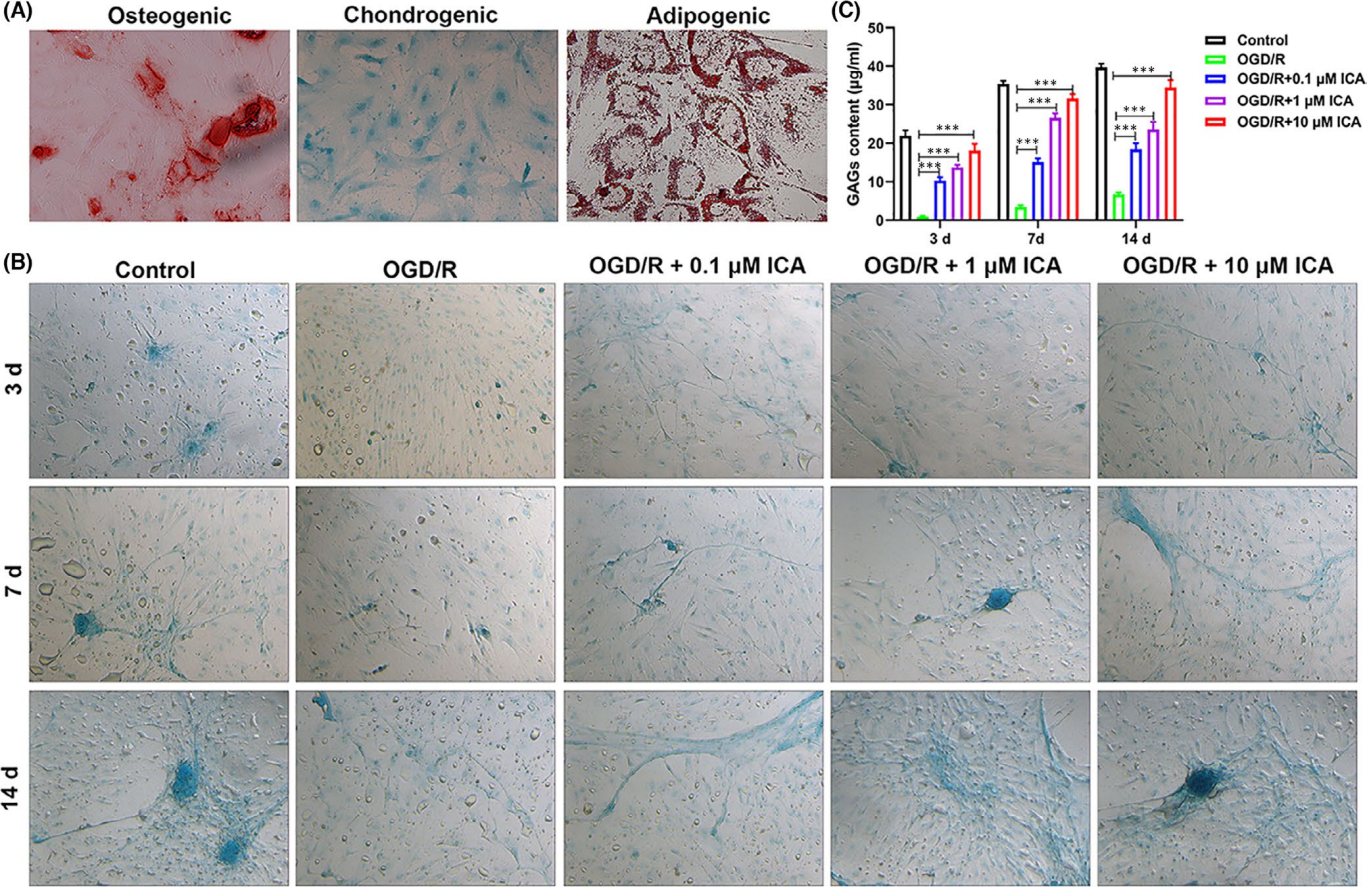
Glycosaminoglycan expression in oxygen‐glucose deprivation/reoxygenation (OGD/R) bone mesenchymal stem cells (BMSCs) at different times as determined by Alcian blue staining. (A) The osteogenic, chondrogenic and adipogenic differentiation levels of BMSCs were detected by Alizarin Red S, Alcian blue and Oil Red O staining respectively. (B and C) Glycosaminoglycan expression and quantitative analysis in OGD/R BMSCs. Control: BMSCs were cultured in normal chondrogenic differentiation medium in the presence of oxygen; OGD/R: BMSCs were cultured in glucose‐ and serum‐free DMEM in an oxygen‐free environment for 4 h and then reoxygenated in normal chondrogenic differentiation medium; OGD/R + 0.1 µM ICA, OGD/R + 1 µM ICA or OGD/R + 10 µM ICA: 0.1, 1 or 10 µM ICA was added to the medium at 24 h before OGD/R conditions were applied

Mesenchymal stem cells have the potential for multidirectional differentiation and can be induced to differentiate into osteoblasts, chondrocytes and adipocytes under certain conditions in vitro. In this study, BMSCs isolated from rabbit bone marrow were analysed for their multidirectional differentiation potential. Alizarin Red S staining showed that BMSCs cultured under osteogenic differentiation conditions contained red nodules, indicating that the BMSCs had osteogenic differentiation potential (Figure 1A). Moreover, Alcian blue staining showed that BMSCs cultured in chondrogenic differentiation medium expressed glycosaminoglycans, indicating that BMSCs had the ability for chondrogenic differentiation (Figure 1A). Finally, BMSCs cultured for adipogenic differentiation contained lipid droplets that could be stained by Oil Red O, indicating the adipogenic differentiation potential of BMSCs (Figure 1A). The results of Alizarin Red S, Alcian blue and Oil Red O staining revealed that the third‐generation BMSCs isolated from bone marrow had osteogenic, chondrogenic and adipogenic differentiation capabilities.

### 3.2 ICA induced glycosaminoglycan expression in BMSCs

Based on characteristics of the isolated BMSCs, we selected the third‐generation BMSCs isolated from bone marrow for use in further studies. We constructed an OGD/R cell model consisting of BMSCs and BMSCs treated in vitro with 0.1, 1 or 10 µM ICA 24 h in advance to analyse the ability of ICA to repair BMSCs after exposure to hypoxic, sugar‐free and serum‐free conditions. Alcian blue staining results showed that BMSCs cultured in chondrogenic differentiation medium could gradually proliferate over time and express increasing levels of glycosaminoglycans, indicating the chondrogenic differentiation ability of BMSCs in the Control group (Figure 1B). However, BMSCs in the OGD/R group showed lower rates of proliferation and levels of glycosaminoglycan expression than those in the Control group, indicating that hypoxia, sugar‐free and serum‐free treatment had reduced the proliferation and chondrogenic differentiation of those BMSCs (Figure 1B). Furthermore, when BMSCs were treated with ICA, the numbers of BMSCs and their levels of glycosaminoglycan expression were higher than those of BMSCs in the OGD/R group. Furthermore, these effects became more obvious as the time of incubation and ICA concentration increased, indicating that ICA could repair the damage to BMSCs created by hypoxic, sugar‐free and serum‐free conditions and increase the chondrogenic differentiation ability of those BMSCs (Figure 1B). And the quantitative analysis of GAGs is shown in Figure 1 (Figure 1C).

### 3.3 ICA induced the chondrogenic differentiation of BMSCs

**FIGURE 2:**
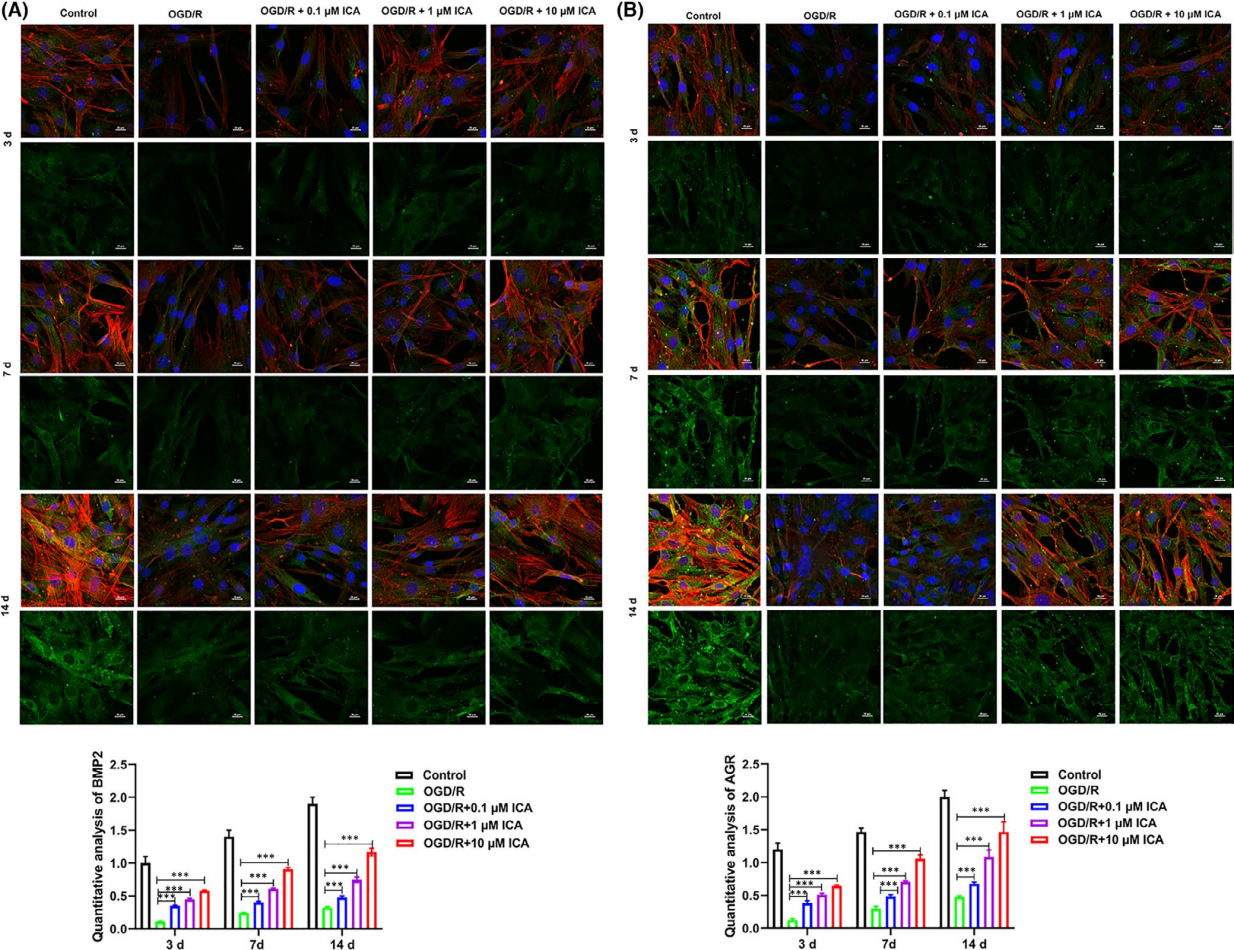
Expression of bone morphogenetic protein 2 (BMP2), aggrecan (AGR) and actin cytoskeleton in oxygen‐glucose deprivation/reoxygenation (OGD/R) bone mesenchymal stem cells (BMSCs) at different times as determined by immunofluorescence. Control: BMSCs were cultured in normal chondrogenic differentiation medium in the presence of oxygen; OGD/R: BMSCs were cultured in glucose‐ and serum‐free DMEM in an oxygen‐free environment for 4 h and then reoxygenated in normal chondrogenic differentiation medium; OGD/R + 0.1 µM ICA, OGD/R + 1 µM ICA or OGD/R + 10 µM ICA: 0.1, 1 or 10 µM ICA was added to the medium at 24 h before OGD/R conditions were applied. Bars represent 20 μm. Blue is DAPI, red is phalloidin, and green is the target protein, (A): BMP2, (B): AGR

**FIGURE 3:**
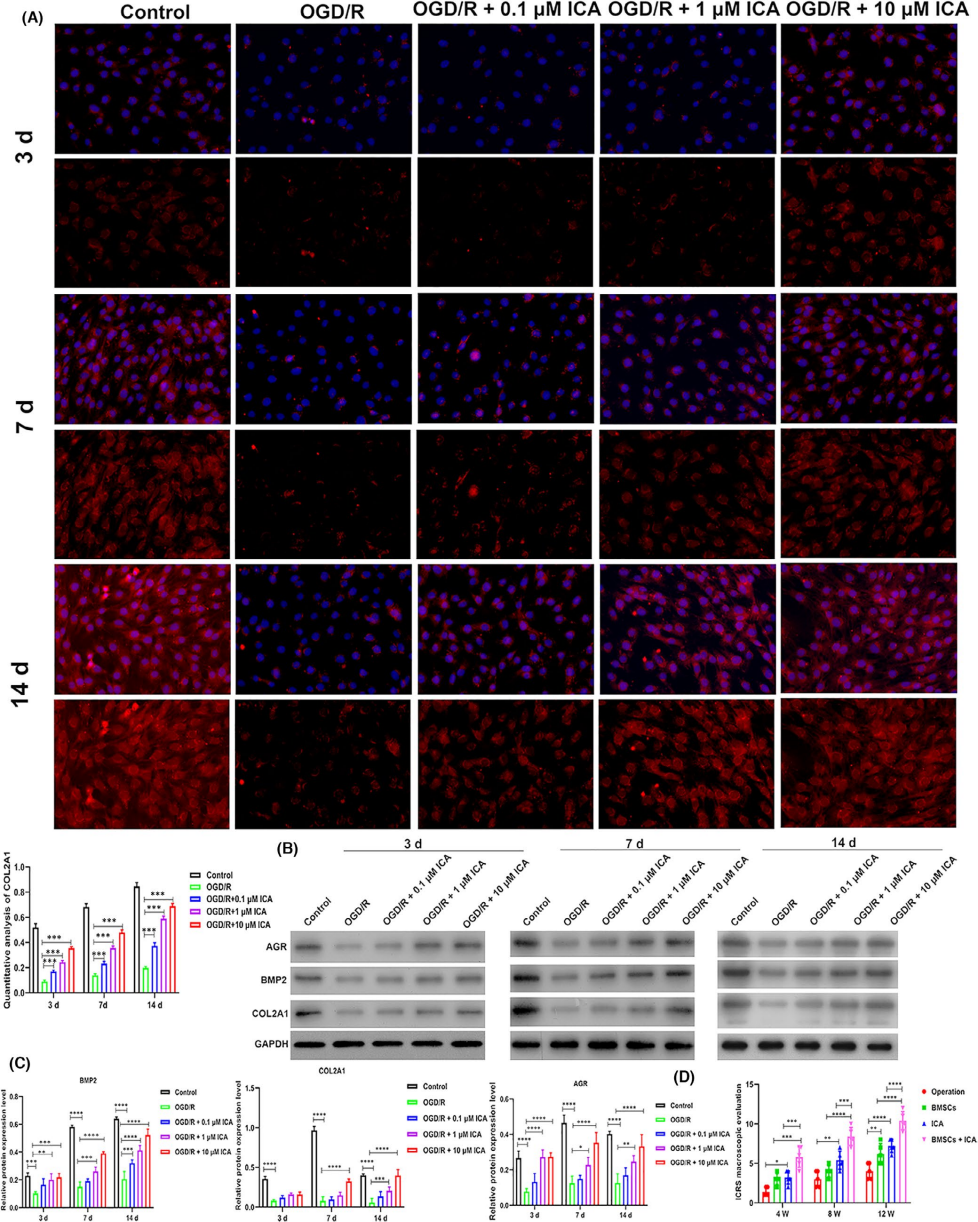
Bone morphogenetic protein 2 (BMP2), collagen type II alpha 1 (COL2A1) and aggrecan (AGR) protein expression in oxygen‐glucose deprivation/reoxygenation (OGD/R) bone mesenchymal stem cells (BMSCs), and the ability of ICA plus BMSCs to repair damaged cartilage. (A) The expression of COL2A1 and actin cytoskeleton in OGD/R BMSCs at different times as determined by immunofluorescence. (B) BMP2, COL2A1 and AGR protein expression at different points was detected by Western blotting. (C) Semi‐quantitative analysis of protein expression levels. (D) The effect of treatment with ICA plus BMSCs on the IRCS rating of cartilage damage at 4, 8 and 12 weeks respectively. Control: BMSCs were cultured in normal chondrogenic differentiation medium in the presence of oxygen; OGD/R: BMSCs were cultured in glucose‐ and serum‐free DMEM in an oxygen‐free environment for 4 h and then reoxygenated in normal chondrogenic differentiation medium; OGD/R + 0.1 µM ICA, OGD/R + 1 µM ICA or OGD/R + 10 µM ICA: 0.1, 1 or 10 µM ICA was added to the medium at 24 h before OGD/R conditions were applied. Operation: normal saline control group; BMSCs: BMSC treatment group; ICA: ICA treatment group; ICA + BMSCs: ICA plus BMSCs treatment group. *p < .05, **p < .01, ***p < .001 and ****p < 0001 represent statistical significance

Based on the above results, we further analysed the ability of ICA to induce cartilage formation by BMSCs. We detected the contents of AGR and COL2A1 in chondrocyte extracellular matrix,^30^ as well as the levels of BMP2, which is one of the most effective factors for inducing the chondrogenic differentiation of mesenchymal stem cells.^31^ Immunofluorescence results showed that the levels of BMP2, AGR, COL2A1 and actin cytoskeleton protein expression and cell proliferation in the OGD/R group were all lower than those in the Control group, indicating that the proliferative and differentiation abilities of BMSCs in the OGD/R group had been reduced (Figures 2 and 3A). This could be explained by the fact that hypoxia, sugar‐free and serum‐free treatment had reduced the proliferation and chondrogenic differentiation of the BMSCs. Furthermore, when BMSCs in the Control group were incubated with ICA, the numbers of cells and their levels of chondrogenic protein expression became upregulated when compared to BMSCs in the OGD/R group (Figures 2 and 3A). Moreover, the ability of ICA to induce chondrogenic‐related protein expression gradually increased in time‐ and concentration‐dependent manners (Figures 2 and 3A). And the quantitative analysis of BMP2, AGR and COL2A1 is shown in Figure 2 (Figures 2 and 3A). In addition, the Western blot results showed that the levels of BMP2, AGR and COL2A1 protein expression in the OGD/R group were significantly reduced when compared with expression in the Control group at 3, 7 and 14 days (Figure 3B,C). Furthermore, when BMSCs were treated with either 0.1 µM or 1 µM ICA, the related protein expression levels in the Control group were higher than those in the OGD/R group at 3, 7 and 14 days (Figure 3B,C). BMSCs treated with 10 µM ICA displayed higher levels of BMP2, COL2A1 and AGR protein expression than BMSCs treated with 0.1 µM or 1 µM ICA and expressed significantly higher levels of those proteins when compared to BMSCs in the ORG/D group at 3, 7 and 14 days (Figure 3B,C). The above results indicated that ICA could induce the chondrogenic differentiation of BMSCs.

### 3.4 ICA and BMSCs combined to repair knee cartilage damage in vivo

Next, ICRS criteria were used to evaluate the repair level of damaged cartilage tissue at 4, 8 and 12 weeks after BMSC and ICA treatment respectively. The mean ICRS score at 4 weeks in the Operation group was 1.4 ± 0.55, which was lower than the mean ICRS score at 4 weeks in both the ICA group (3.2 ± 0.84) and BMSCs group (3.0 ± 0.71), indicating that the cartilage in the rabbit knee joint was severely abnormal, and had not obviously improved after BMSC or ICA treatment (Figure 3D). However, the mean ICRS score in the BMSC + ICA group at 4 weeks was 5.8 ± 1.3, which was significantly higher than the mean ICRS score in both the BMSCs group (*p* < .001) and ICA group (*p* < .001), suggesting that the extremely damaged cartilage was partially repaired after BMSC plus ICA treatment at 4 weeks (Figure 3D). The mean ICRS scores at 8 weeks in the Operation, BMSCs, ICA and BMSCs + ICA groups were 3.0 ± 1.0, 4.0 ± 0.71, 5.4 ± 1.1 and 8.4 ± 1.1, respectively, indicating that the damaged cartilage had improved when compared to cartilage in the ICA or BMSCs treatment groups at 4 weeks (Figure 3D). Furthermore, at 12 weeks, the ICRS scores in the Operation, BMSCs, ICA and BMSCs + ICA groups were 4.0 ± 1.0, 6.2 ± 1.3, 7.2 ± 0.84 and 10 ± 1.1 respectively (Figure 3D). These data indicated that the articular cartilage in the BMSCs and ICA groups was still abnormal, but was nearly normal in the BMSC + ICA group at 12 weeks (Figure 3D). The above results showed that while either BMSC or ICA treatment alone could repair damaged cartilage tissue, the effect of combining treatment with BMSC plus ICA was better than the effect produced by either treatment alone and also showed a time‐dependent therapeutic effect.

### 3.5 ICA and BMSCs combined to affect the pathological changes and proliferation of damaged cartilage tissue

**FIGURE 4:**
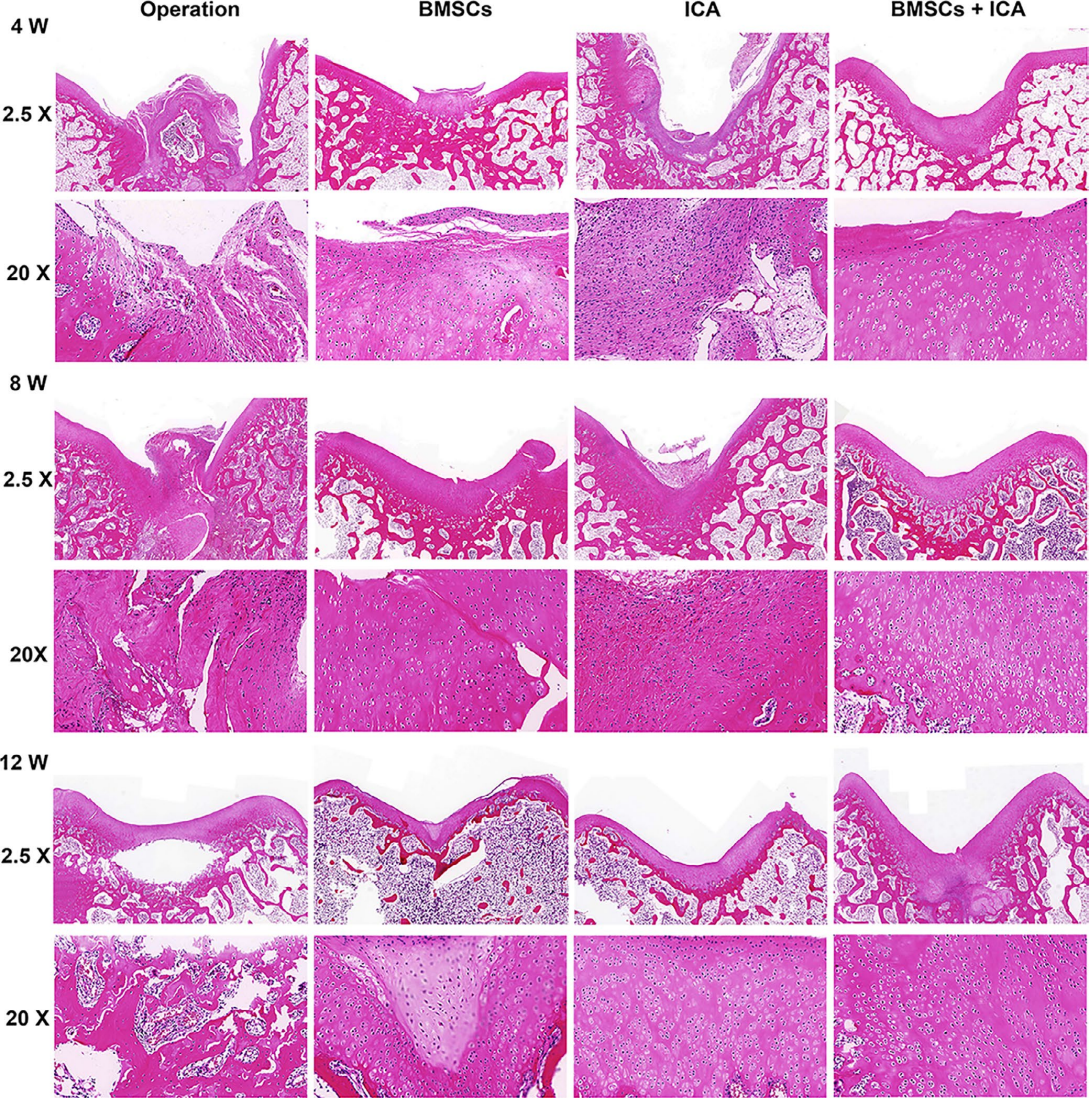
Haematoxylin‐eosin (H&E) analysis of damaged cartilage tissue at 4, 8 and 12 weeks after treatment with ICA plus bone mesenchymal stem cells (BMSCs). Operation: normal saline control group; BMSCs: BMSCs treatment group; ICA: ICA treatment group; ICA + BMSCs: ICA combined with BMSCs treatment group. Bars represent 500 μm

**FIGURE 5:**
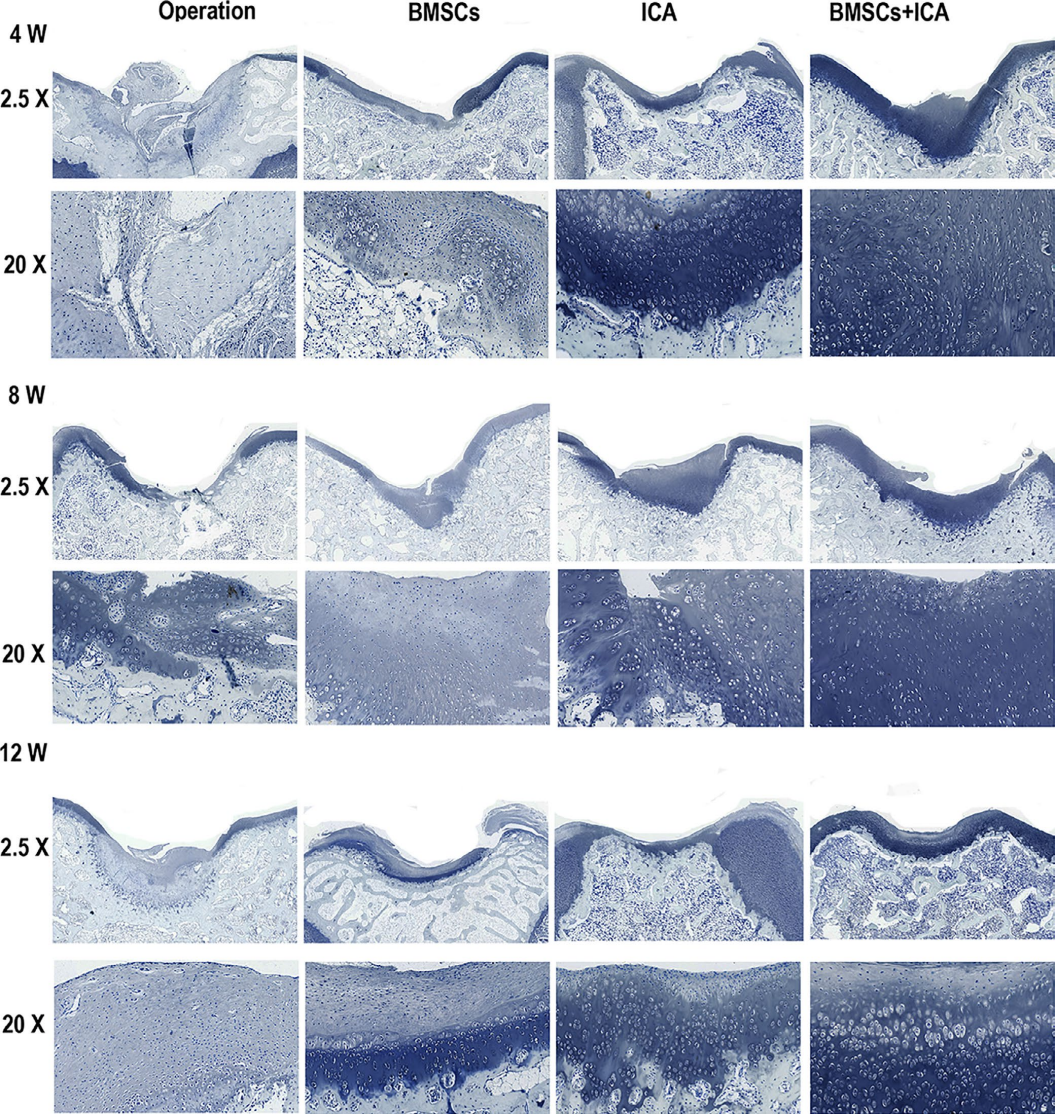
Toluidine blue staining analysis of damaged cartilage tissue at 4, 8 and 12 weeks after treatment with ICA plus bone mesenchymal stem cells (BMSCs). Operation: normal saline control group; BMSCs: BMSCs treatment group; ICA: ICA treatment group; ICA + BMSCs: ICA plus BMSCs treatment group. Bars represent 500 μm

**FIGURE 6:**
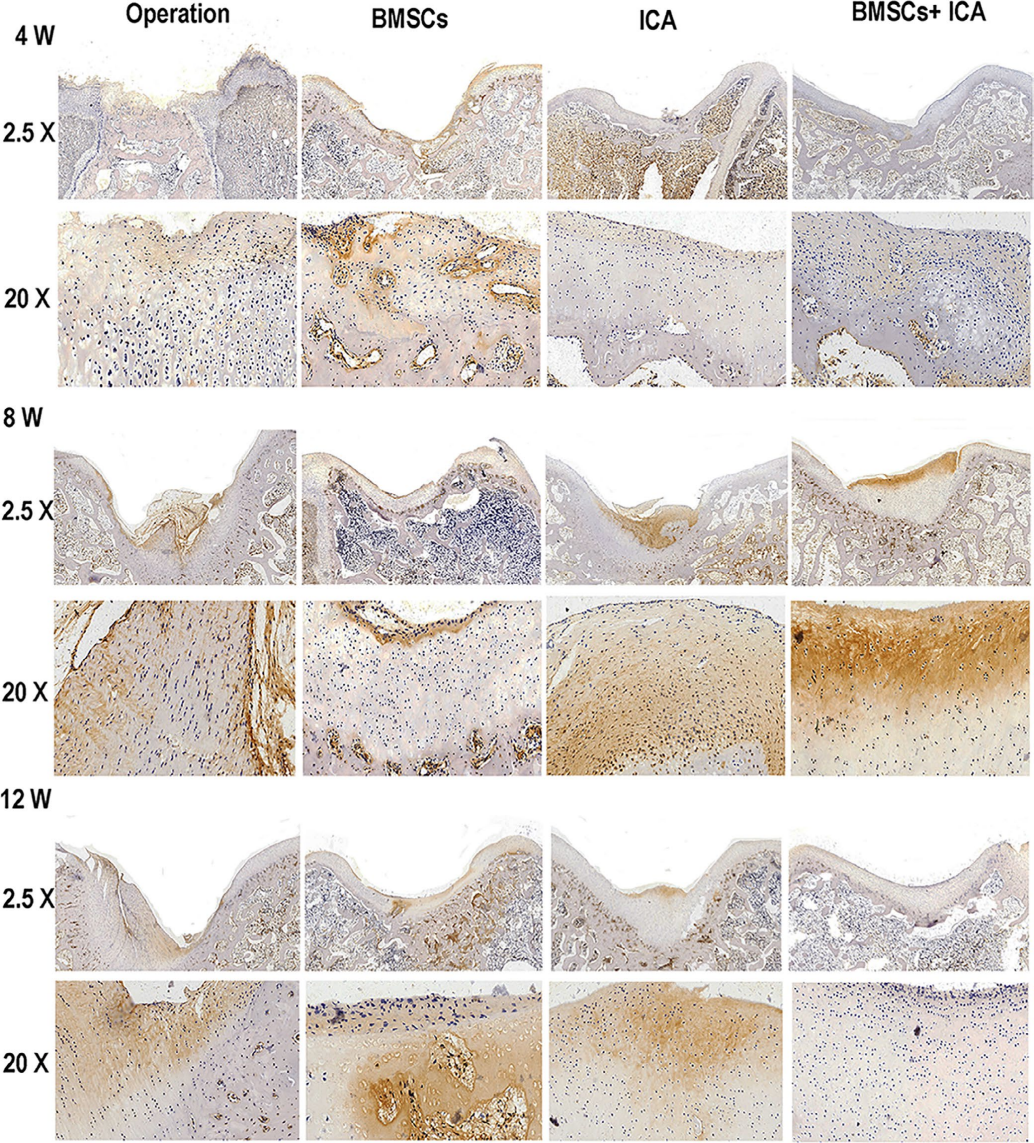
Immunohistochemistry analysis of damaged cartilage tissue at 4, 8 and 12 weeks after treatment with ICA plus bone mesenchymal stem cells (BMSCs). Operation: normal saline control group; BMSCs: BMSCs treatment group; ICA: ICA treatment group; ICA + BMSCs: ICA plus BMSCs treatment group. Bars represent 500 μm

**FIGURE 7:**
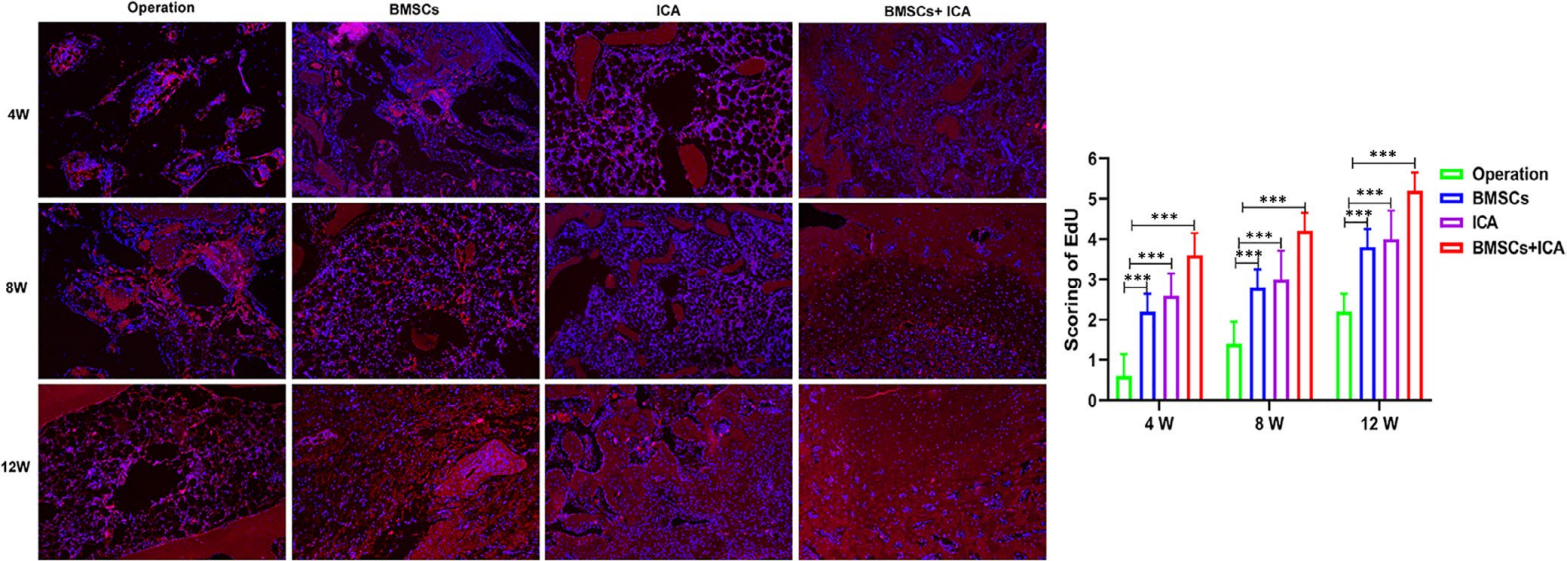
Effect of combined ICA and bone mesenchymal stem cell (BMSC) treatment on the proliferation of damaged cartilage tissue as determined by EdU staining at 4, 8 and 12 weeks respectively. Operation: normal saline control group; BMSCs: BMSCs treatment group; ICA: ICA treatment group; ICA + BMSCs: ICA plus BMSCs treatment group

We also observed the pathological features of damaged cartilage tissue after 4, 8 and 12 weeks of treatment. H&E staining results revealed an irregular cartilage surface, cracks, narrowing of the cartilage layer and severely damaged cartilage structure in the Operation group at 4 weeks (Figure 4). After either ICA or BMSC treatment alone, the damaged cartilage tissue had not significantly improved at 4 weeks; however, the cracks in the cartilage injury area became narrower and the cartilage layer became thicker after 4 weeks of combined treatment with BMSCs plus ICA (Figure 4). As the time was extended to 12 weeks, the combined treatment with BMSCs and ICA produced a smooth surface and integrated cartilage structure in the damaged cartilage tissue (Figure 4). In addition, toluidine blue staining results showed that either ICA or BMSC treatment could upregulate the content of extracellular matrix (ECM) when compared to its content in the Operation group (Figure 5). After ICA plus BMSC treatment, the ECM was heavily stained with toluidine blue, and the staining intensity gradually increased with the prolongation of time up to 12 weeks (Figure 5). Immunohistochemistry results showed that either ICA or BMSC treatment alone could significantly increase the COL2A1 content of damaged cartilage tissue (Figure 6). After treatment with combined ICA and BMSCs, the yellow‐stained area increased and COL2A1 expression increased in a time‐dependent manner (Figure 6). Moreover, EdU staining results showed that either ICA or BMSC treatment could enhance the proliferation of chondrocyte cells, and combined treatment produced a stronger and time‐dependent therapeutic effect (Figure 7). The above results indicated that treatment with ICA plus BMSCs could repair damaged cartilage tissue in a time‐dependent manner.

## 4 DISCUSSION

Articular cartilage, which covers the surface of joints, is composed of cartilage cells, cartilaginous ECM and water.^32^ Cartilaginous ECM is mainly composed of collagen, ARG and glycosaminoglycan.^30^ These components play an important role in buffering stress, absorbing shock, lubricating joint surfaces and preventing wear.^32^, ^33^ However, once the articular cartilage is damaged, it causes pathological changes in the joints and leads to osteoarthritis, thus affecting a person’s quality of life.^34^ Therefore, it is essential to seek effective methods for treating articular cartilage damage. In this study, ICA and BMSCs were selected to explore their effects on knee cartilage injuries. Our results showed that either ICA or BMSCs alone could repair cartilage injuries in the rabbit knee. Moreover, combined treatment with ICA plus BMSCs was even more effective for repairing rabbit knee cartilage defects, and the therapeutic effect increased with time up to 12 weeks.

Mesenchymal stem cells can differentiate into chondrocytes, osteocytes and adipocytes, which makes them an excellent choice for use in cell therapy.^35^ There have been numerous reports that BMSC implantation can be used to treat patients with osteoarthritis, and hyaline cartilage‐like tissue has been identified at a defect site at 42 weeks after implantation.^36^ BMSC transplantation also increases the differentiation of chondrocytes and expression of type II collagen and suppresses bone destruction in arthritic rats.^37^ However, the survival rate of transplanted stem cells decreases in an ischaemic and hypoxic environment, and this decrease seriously affects the repair efficiency of stem cells.^38^ Interestingly, ICA relieves OGD‐induced rabbit BMSC damage by promoting BMSC proliferation and repressing BMSC apoptosis.^24^ This suggests that ICA might promote BMSC proliferation in hypoxic and ischaemic cartilage tissue. In our research, an OGD/R BMSC model was established. Simultaneously, the model cells were treated with different concentrations of ICA, and results showed that ICA promoted the chondrogenic differentiation of BMSCs, as well as the expression of AGR, BMP2 and COL2A1 proteins in BMSCs. The above results indicated that ICA repaired the damage caused by hypoxia and glucose deficiency in BMSCs and also induced the expression of cartilaginous ECM in BMSCs.

Previous research showed that mechanical stress can lead to chondrocyte apoptosis and the destruction of cartilage tissue. Moreover, high levels of chondrocyte apoptosis have been reported in osteoarthritis tissue.^39^ ICA treatment reduces cartilage destruction, enhances chondrocyte differentiation, and significantly reduces cartilage degeneration in osteoarthritis.^40^ ICA can also downregulate the levels of chondrocyte matrix metalloproteinase‐3, metalloproteinase‐9 and metalloproteinase‐13 mRNA expression, decrease the levels of disintegrin and metalloproteinase with thrombospondin motifs type 4 (ADAMTS‐4), promote the synthesis of type II collagen and glycosaminoglycan and reverse the imbalance of ECM homeostasis caused by interleukin 1β; these effects allow ICA to play a protective role in chondrocytes.^41^ Consistently, our results showed that when compared to the Operation group, ICA treatment resulted in a higher mean ICRS score, restored cartilage‐like tissue and enhanced COL2A1 expression on the defect surface. In addition, compared with ICA treatment, BMSC exhibited the similar protective effect against cartilage damage. It is also consistent with previous studies that demonstrate the promoting effects of BMSCs on the regeneration of bone and cartilage. Notably, we found that combined treatment with ICA and BMSCs produced an even better recovery of cartilage damage. It can be explained that ICA not only exerts protective role in preventing cartilage destruction, but also promotes chondrogenic differentiation of BMSCs, which further enhance the recovery of cartilage damage. However, the potential mechanism for that recovery from cartilage damage remains to be explored.

## 5 CONCLUSION

In conclusion, treatment with ICA or BMSCs alone was shown to repair rabbit knee cartilage damage. Moreover, treatment with a combination of ICA and BMSCs demonstrated an even better ability to repair rabbit knee cartilage defects, which provides a certain basis for the treatment of osteoarthritis.

## CONSENT FOR PUBLICATION

Consent for publication was obtained from all authors.

## CONFLICTS OF INTEREST

All authors declare having no conflicts of interest related to this research.

## AUTHOR CONTRIBUTIONS

**Wang Tang:** Data curation (lead); Investigation (lead); Methodology (lead); Writing—original draft (lead). **Hongyi Zhang:** Methodology (supporting); Project administration (lead); Visualization (supporting). **Donghua Liu:** Investigation (supporting); Supervision (lead); Writing—original draft (supporting). **Feng Jiao:** Funding acquisition (lead); Methodology (lead); Writing—review & editing (lead).

## Data Availability

All data generated or analysed during this study are included in this published article.
